# Research on strength and microscopic characteristics of lime-activated fly ash-slag solidified sludge under high temperature effect

**DOI:** 10.1371/journal.pone.0305761

**Published:** 2024-06-18

**Authors:** Shunmei Gong, Songbao Feng, Shiquan Wang, Lemei Yu, Yuanyuan Chen, Qiang Xu, Zhiyong Niu

**Affiliations:** 1 National Engineering Research Center of Coal Mine Water Hazard Controlling, School of Resources and Civil Engineering, Suzhou University, Suzhou, China; 2 Jiangsu Weixin Engineering Consulting Co., Ltd., Nanjing, Jiangsu, China; Amirkabir University of Technology (Tehran Polytechnic), ISLAMIC REPUBLIC OF IRAN

## Abstract

To explore the reaction mechanism of sludge, slag, lime, and fly ash in high temperature environments, the unconfined compressive strength (UCS) test was hereby implemented to study the effect on curing age, curing temperature, slag content and fly ash content about the strength of sludge. Scanning electron microscopy (SEM) was used to observe the microscopic composition of the substance, and X-ray diffraction (XRD) was used to analyze the mineral composition at the micro level to further disclose its reinforcement mechanism. The experimental results demonstrate the difference in the strength measured by different dosage of curing agent, and results indicate that the strength of high temperature curing sample was obviously higher than that of low temperature curing sample. When the curing temperature rises, the pozzolanic reaction and hydration reaction between materials are accelerated, and a certain amount of gel products are produced, playing a precipitation and bonding role between particles. The 28 days and 90 days strengths of the sludge samples with 20% fly ash and 80% slag dosing at 40°C were 1139 KPa and 1194 KPa, which were 1.4 and 1.1 times of that of pure cement solidified sludge. At 60°C, the strength of 14 days, 28 days and 90 days were 802 KPa, 1298 KPa and 1363 KPa, which were 1.1, 1.5 and 1.3 times of that of pure cement solidified sludge. Under the influence of an alkaline environment, the silicon-aluminum grid structure was interconnected into a denser network structure, and the compressive strength of lime-activated fly ash-slag was thus continuously enhanced. Affected by the high temperature, lime-activated fly ash-slag solidified sludge could significantly improve the middle and late strength of the sample. The research showed that the new solidification material can replace partly the concrete curing agent, thereby alleviating the carbon emission and environmental pollution problems arising from cement solidified sludge.

## Introduction

A lot of dredged sludge is produced in the process of urban water environment treatment. The effective treatment of dredged sludge can solve the scarcity of state-owned land resources. However, in the traditional process of dredging sludge solidification, curing agents such as lime and cement were generally used, which, though effectively reinforcing the soft soil foundation, still discharges a large amount of CO_2_ gas, causing serious air pollution problems. In this case, the development of a green and sustainable curing agent has become a research hotspot in the field of soft soil foundation reinforcement [1–3].

The new curing agents developed by scholars at home and abroad are mainly industrial by-products, and considerable research results have been achieved. Meanwhile, granulated blast furnace slag (referred to as slag) and fly ash are also widely used in curing agent raw materials [4]. Since the market price of fly ash is 50% that of cement, and the market price of slag is 30% that of cement, the engineering application value of industrial waste can be improved, and the environmental pollution of traditional curing agent can be reduced once alkali-activated fly ash-slag can partially substitute concrete curing agent. At standard indoor curing temperature, Dassanayake KB et al. [5] has been discovered that fly ash could increase the strength of solidification sludge based on experiments. Wang DX et al. [6] confirmed that fly ash and active MgO could improve the overall mechanical properties of solidified sludge by water stability test. Jin F et al. [7] conducted microscopic experiments and found that the hydration reaction product of the MgO-slag system was mainly hydrated calcium silicate, and that the hydration product filled the gap between the particles and improved the strength of the solidified sludge. Indeed, in practical engineering practice, a big difference can be observed between the indoor test environment and the on-site sludge solidification environment, and the ambient temperature was a related factor influencing the mechanical character of soil [8]. The actual temperature of the soil was more than the environmental temperature because of the hydration heat release during the sludge solidification process [9, 10]. Al Mukhtar [11, 12] compared the characteristics of lime improved soil under curing conditions of 20°C and 50°C, and found that the pozzolanic reaction was accelerated and the UCS of improved sludge increased rapidly under curing conditions of 50°C. Wang DX [13] proved through experiments that the activity of slag and fly ash could be improved with the increase of curing temperature. Okoye F N et al. [14] confirmed through experiments that the addition of industrial waste can significantly improve the strength of concrete. Kang X et al. [15] proved that the combined action of lime and fly ash can improve the stability of pavement base. Gong SM et al. [16] studied the effect of different curing temperatures on the new slag mixture. The results confirmed that the curing temperature can improve the strength of solid waste solidified sludge. Fly ash has a positive effect on the strength of macropores in solid mixtures [17–19]. Balendran RV [20] researched the characteristics of fly ash mixed sludge at higher temperatures, and discovered that fly ash could partially replace cement and increase its compressive strength by 15%. Zhang RJ [21] discovered that high temperature was able to obviously improve the speed of chemical reaction between clay, fly ash and slag, and produced a certain amount of C-S-H gel products. By studying the strength of marine soft soil, Phetchuay C [22] discovered that when the curing temperature ascended to 40°C, that the geopolymerization reaction between soil, fly ash and slag was further enhanced, and that the long-term strength of solidified soil was improved. Liu MD et al. [23] proved that the strength of solidified soil is affected by curing temperature, and there is a certain change rule between them. Consoli NC et al. [24] discovered that from the point of view of sand modifier, the increase of temperature accelerated the reaction rate between rice husk ash and carbonized slag, and that the strength of the sample cured at 40°C for 7 days was greater than that cured at 20°C for 7 days. The above literature has shown that the reaction rate between curing agent and solidified sludge is related to temperature, but the evolution of mechanical behavior of solidified sludge induced by high temperature environment in practical engineering practice has been rarely reported, especially while considering the high temperature effect. Also, there are few reports that the mixed curing agent of fly ash and slag is activated by lime. To this end, the lime excited fly ash-slag curing agent and its reaction mechanism as sludge curing agent were studied under high temperature effect, the influence of fly ash content, slag content, curing temperature and curing age on the strength development law of cured silt was hereby explored, and compared with that of cement solidified sludge samples. It should be noted that the impact of high temperatures on the stimulation of pozzolanic reactions and hydration reactions in fly ash-slag, specifically under the influence of lime, poses a challenging question. Exploring whether this combination can generate gel material to effectively cement soil particles is a complex issue that warrants further investigation in future research endeavors.

## Materials and methods

### Materials

The experimental sludge was obtained from the dredging site of a river bottom tunnel in Xuzhou City. The following Table 1 is the basic properties of sludge. The initial water content of the waste sludge was 50%, and the main components were clay, sludge and fine sand, accounting for 19%, 38% and 43%, respectively. The lime in the test material was purchased from a lime sales company in Xuzhou City. The appearance was light gray powder, and CaO accounted for 84.88% as the main component. The fly ash in the test material was purchased from a steel plant in Xuzhou City. The fly ash used in the experiment was purchased from a steel plant in Xuzhou City. The appearance was gray powder, and the main components SiO_2_ and Al_2_O_3_ accounted for 53.26% and 26.53%, respectively. The slag involved was purchased from a slag processing plant in Xuzhou City. The appearance was gray-white powder, and CaO and SiO_2_ accounted for 40.79% and 31.99%, respectively as the main components. The following Table 2 is the composition of the sludge. The following Table 3 is the composition of raw materials.

**Table 1:** Basic properties of sludge.

Relative density/(g/cm^3^)	Initial water content /%	Optimum moisture content /%	Liquid limit /%	Plastic limit /%	Composition		
					Clay /%	powder particle /%	Fine sand /%
2.68	50	20.25	53.85	31.24	19	38	43

**Table 2:** Chemical composition of the sludge.

Silicate (%)	Carbonate (%)	Sulfate (%)	Organic matter (%)	Others (%)
49.28	36.73	7.95	5.01	1.03

**Table 3:** Chemical composition of raw materials.

Chemical composition	Raw material content /%		
	Lime	Fly ash	Slag
**CaO**	84.88	2.45	40.79
**Al**_**2**_ **O**_**3**_	2.24	26.53	11.85
**Fe**_**2**_ **O**_**3**_	0.33	3.25	0.68
**MgO**	6.14	4.19	5.95
**SiO**_**2**_	3.15	53.26	31.99
**CaSO**_**4**_	1.16	8.59	1.47
**others**	2.10	1.73	7.27

### Test scheme

Based on the test scheme of Wang SN [25] and Zhang M [26], a total of 7 groups of samples with different mix ratio parameters were set up, and 3 parallel samples were established under different curing conditions. Lime made for 20% of the sludge’s total 10% curing agent concentration in the experiment, and the remaining 80% was fly ash and slag. A set of comparative samples was established using a pure cement curing agent. Table 4 below listed the seven types of curing agent ratio schemes that were in place. In order to facilitate the expression, Symbol *F*_*x*_*G*_*y*_ represented the various ratios of fly ash and slag, In the formula, *F* represents fly ash, *G* represents slag, *X* and y represent different percentages. ce represents cement. The curing temperatures of lime-excited fly ash-slag solidified sludge samples were set to 20°C, 40°C and 60°C. The sludge that had solidified with cement had a temperature of 20°C. The curing ages were 3, 7, 14, 28, and 90 days, and the lime-fly ash-slag samples with different ratios were compared with the cement solidified sludge samples.

**Table 4:** Test scheme.

Group number	Lime content /%	Fly ash:Slag	Cement dosage /%	Curing temperature /°C	Curing age /d
F10G0	20	10:0	0	20、40、60	3、7、14、28、90
F8G2		8:2			
F6G4		6:4			
F4G6		4:6			
F2G8		2:8			
F0G10		0:10			
ce	0	0	10	20	

### Test methods

The strength of the sample was measured with reference to the *Standard for Soil Test Methods* (GB/ T 50123–2019) [27]. The strength test of the samples in this paper were tested by universal testing machine, and the loading rate of 2 mm/min was set. Three parallel samples were set up under different experimental conditions to guarantee the experiment’s precision and repeatability. Besides, the average value obtained was taken as the final strength of the sample. The microstructure of the sample was observed by SEM, and the sample after grinding was scanned using XRD. The scanning angle was between 5–80° and 5°/min was the scanning rate of the sample.

## Results and discussion

### Unconfined compressive strength (UCS)

#### Effect on curing temperature and curing age on the process of UCS development of the sample

**Fig 1:**
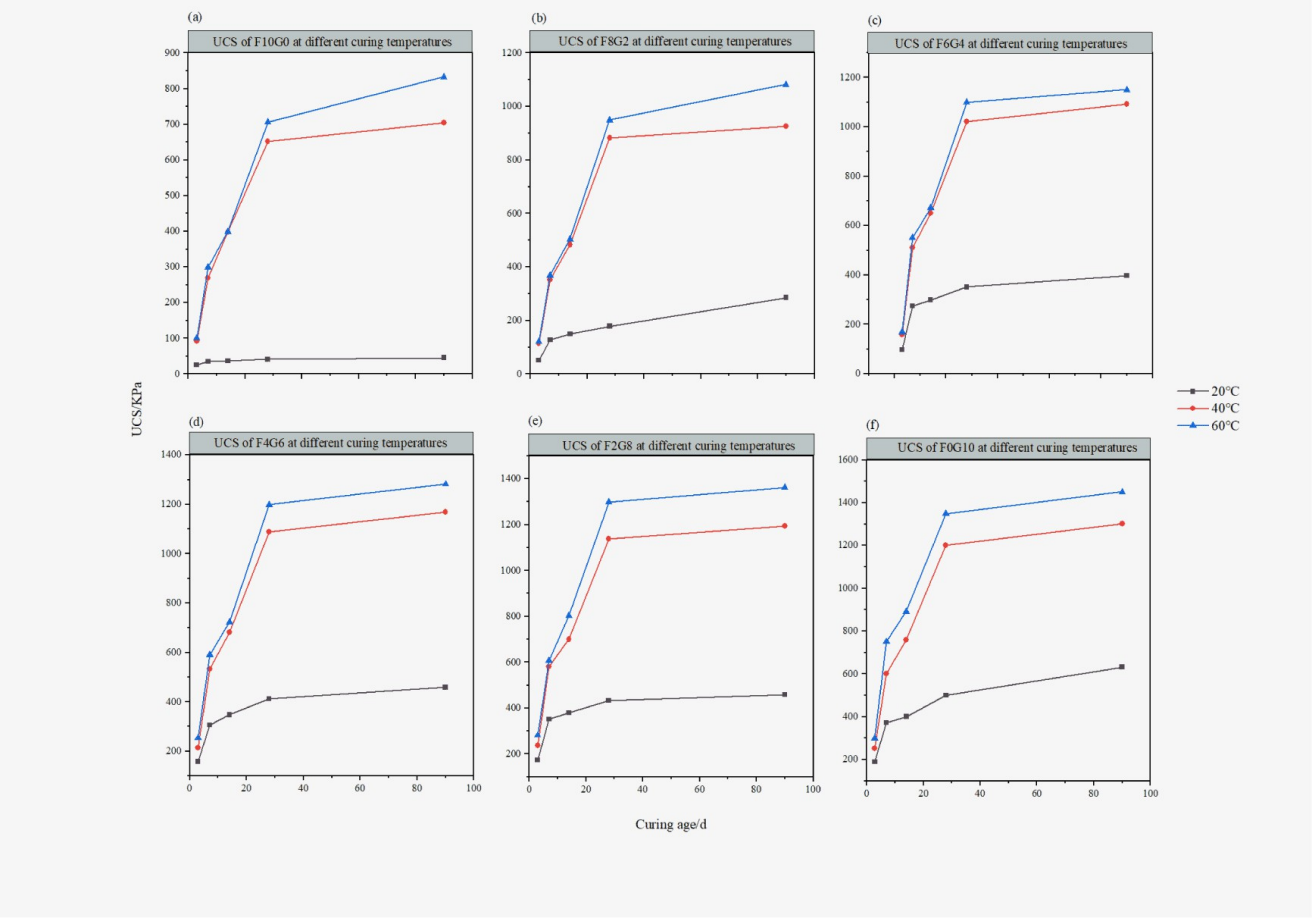
UCS of different samples at different curing ages. The UCS of each sample is obtained by the average of three parallel samples. (data presented in S1 Table in S1 File).

At the curing temperature of 20°C, 40°C and 60°C, the development of UCS of lime-fly ash-slag solidified sludge samples with different ratios with age is shown in Fig 1(A)–1(F). At the same curing temperature, the UCS of each sample presented a similar change rule, that is, the UCS of the sample showed an increasing trend as the curing age increased. The change rules of samples with different proportions were quite different. At the curing temperature of 20°C, the growth trend of sample F10G0 was less affected by curing age. From a macro perspective, fly ash is considered an inert substance, and room temperature curing fails to improve the reaction between its internal substances. At the curing temperature of 20°C, the growth rate of other sample was relatively fast at the initial stage of curing (7~14 days), which was then relatively slow. From a macro point of view, the increase of slag ratio could be improved the early UCS of the sample. Under different curing agent contents and component mix ratios, the UCS of the sample was greatly affected by the curing temperature. When the sample was maintained to 28 days, the UCS growth rate of high temperature curing reached its maximum, and then, in the process of curing age reaching 90 days, the strength increasing of the sample gradually slowed down. Taking an example of F8G2, at 20°C, the compressive strengths of lime-activated fly ash-slag solidified sludge at 3, 7, 14, 28, and 90 days were 50 KPa, 127 KPa, 149 KPa, 178 KPa, and 285 KPa, respectively, while at 40°C, the compressive strengths of lime-activated fly ash-slag solidified sludge at 3, 7, 14, 28, and 90 days were 114 KPa, 352 KPa, 482 KPa, 882 KPa and 926 KPa, respectively. Then, at 60°C, the compressive strengths of lime-activated fly ash-slag solidified sludge at 3, 7, 14, 28, and 90 days were121 KPa, 369 KPa, 503 KPa, 949 KPa and 1082 KPa. The experimental results showed that the strength of the low temperature curing sample is lower than that of the high temperature curing sample.

It was further found through experiments that lime could stimulate the pozzolanic reaction and hydration reaction between sludge, slag and fly ash in high temperature environments. When using lime as an activator, lime hydrated with water to form calcium hydroxide, facilitating alkaline conditions for chemical reactions. The silica and alumina in fly ash accounted for about 80%, and the calcium oxide in slag accounted for about 85%. Under alkaline conditions, hydroxide ions reacted with silica and other substances to form a rich *C*−*S*−*H* gel. Part of the fly ash and slag were eroded to release free silicon-aluminum substances, which then reacted with *C*a^2+^ and *SO*_4_^2−^ to form needle-like ettringite. The gel generated by the chemical reaction filled the gap between the particles, and the overall strength of solidified sludge was improved. The chemical reaction is shown in Formula 1.


$$\begin{array}{l} CaCO_{3}+H_{2}O\to Ca{\left(\mathrm{OH}\right)}_{2}+{\mathrm{CO}}_{2}\\ SiO_{2}+Ca^{2+}+2OH^{-}\to CaO\cdot SiO_{2}\cdot H_{2}O(C-S-H)\\ Al_{2}O_{3}+Ca^{2+}+OH^{-}\to CaO\cdot Al_{2}O_{3}\cdot H_{2}O(C-A-H)\\ Al_{2}O_{3}+Ca^{2+}+OH^{-}+S{O_{4}}^{2-}\to 3CaO\cdot Al_{2}O_{3}\cdot 3CaSO_{4}\cdot 32H_{2}O(C-A-S-H) \end{array}$$ (1)

#### Effect of curing agent content on the process of the sample strength development

**Fig 2:**
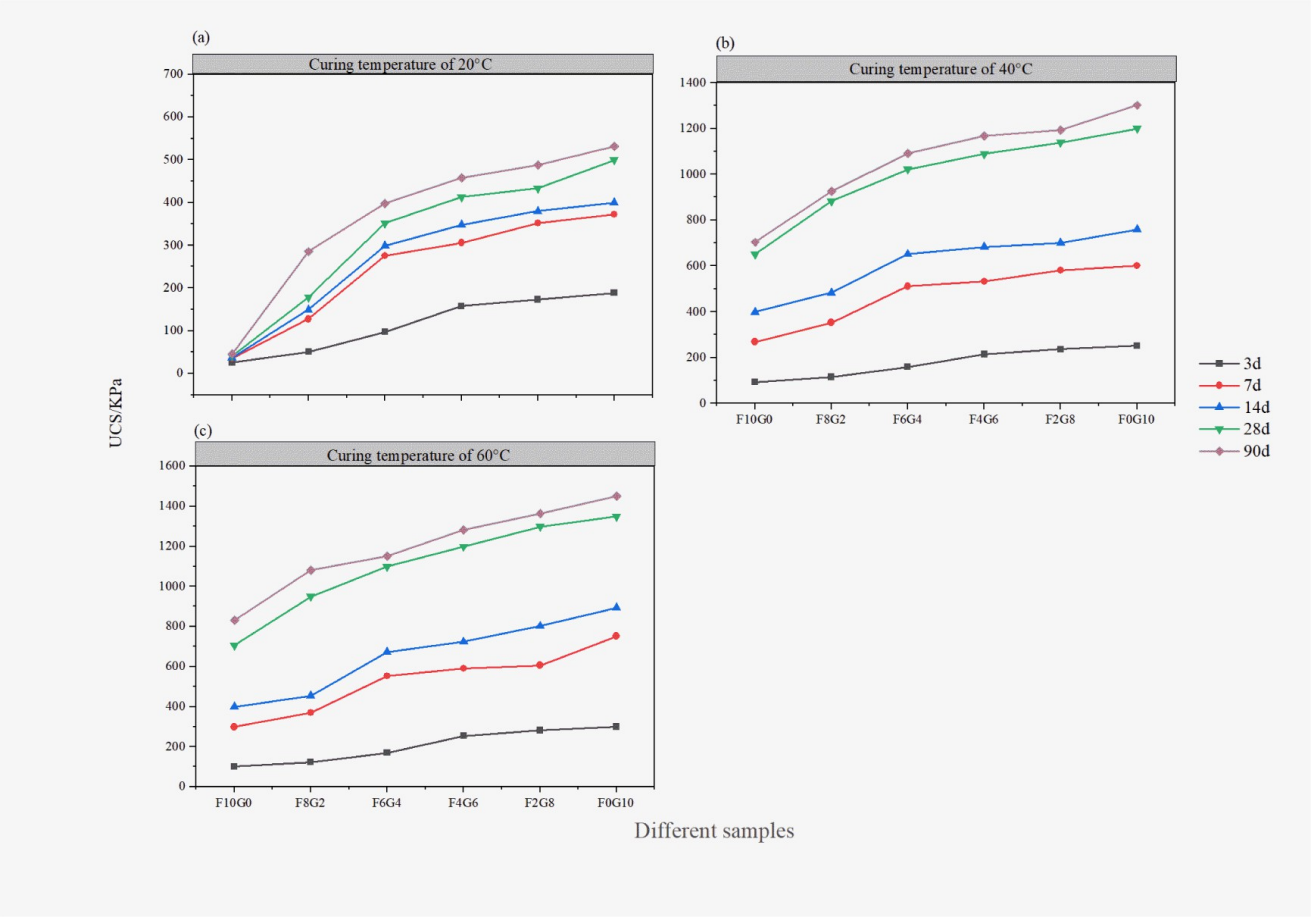
The effect of curing agent content on the strength of sludge samples. At different curing temperatures, the UCS of the sample is obtained by the average value of the three parallel samples. (data presented in S2 Table in S1 File).

Fig 2(A)–2(C) present the dependence curves between the content of solidified material and strength at curing ages of 3, 7, 14, 28 and 90 days under different temperature conditions. Observing the law of the following figure, under the same curing temperature conditions, with the increase of slag ratio and curing age, the strength showed an increasing trend. The UCS of sample F4G6 with curing period of 28 days and curing temperature of 20°C, 40°C and 60°C were 412 KPa, 1088 KPa and 1198 KPa, respectively, while that of sample F4G6 with curing period of 90 days and curing temperature of 20°C, 40°C and 60°C were 458 KPa, 1169 KPa and 1282 KPa, respectively. Meanwhile, the 28-day strength and 90-day strength of the sample were relatively close, indicating that the engineering age could be further shortened with higher temperature. Considering the economic cost, the compressive strength of sample F4G6 reached the best value of this test, which shortened the construction period and realized the purpose of energy saving, environmental protection and green low carbon. According to the analysis, the main components of lime and slag had undergone hydration reaction, and a large amount of hydroxyl ion been generated, providing an alkaline environment for the reaction between substances, and stimulating the activity of SiO_2_ and Al_2_O_3_ in inert fly ash. In this case, C-S-H were generated. While filling the voids, the soil particles and other products could be cemented to improve the UCS of the sludge.

#### Comparative analysis

**Fig 3:**
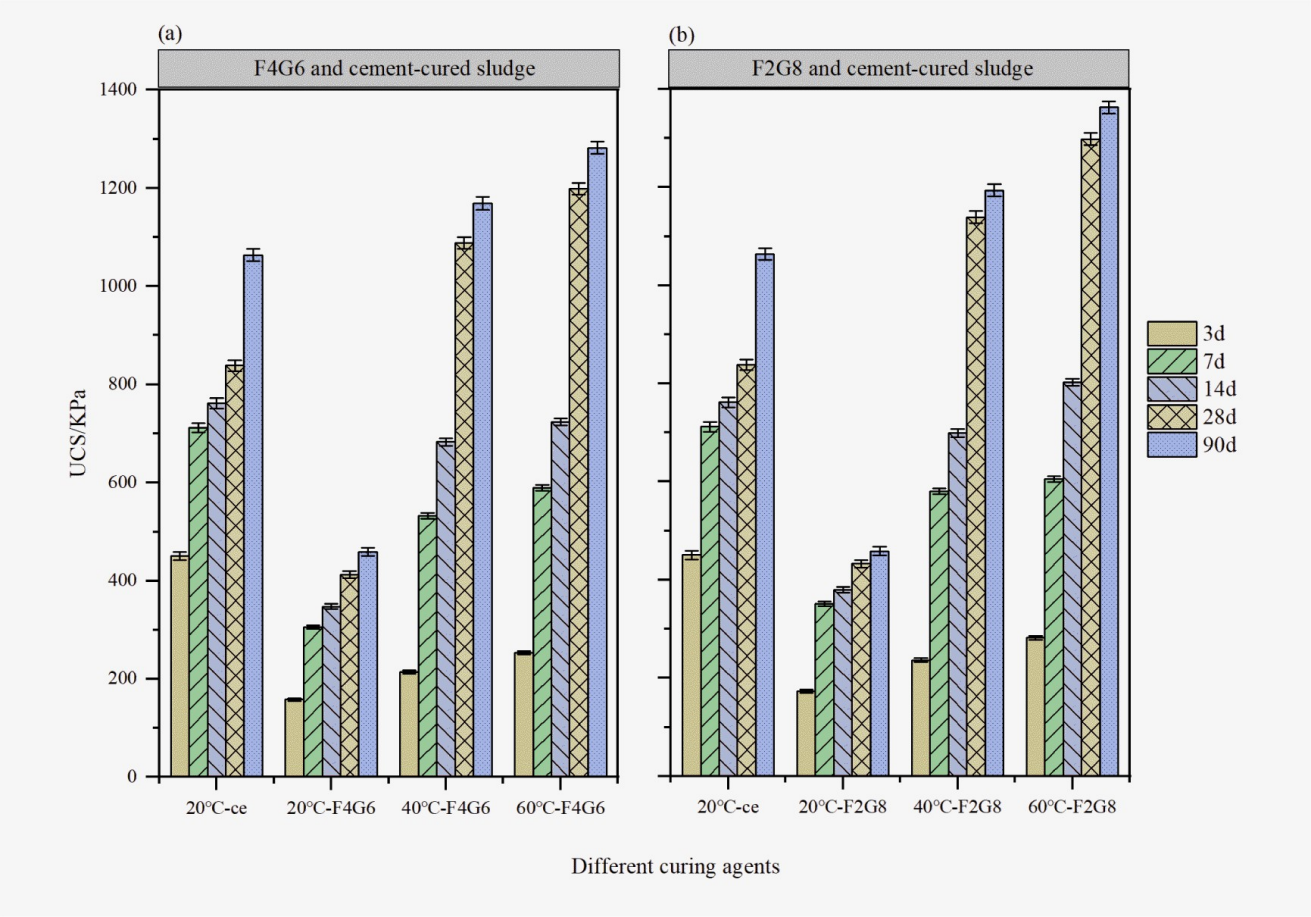
Comparison of compressive strength of representative samples with cement-cured sludge under different curing temperature. The error bar represents the standard deviation of three repetitions. The UCS of the sample under different temperature curing is obtained by the average of the three parallel samples. (data presented in S3 Table in S1 File).

In order to reinforce industrial waste and replace cement curing agent using the waste heat of blast furnace, a systematic experiment on lime-activated fly ash-slag solidified sludge under high temperature effect was carried out through UCS experiment, and a parallel comparison test was conducted with cement solidified sludge under 20°C curing condition. Taking samples F4G6 and F2G8 as an example, as shown in Fig 3(A) and 3(B), under the condition of 20°C curing, the compressive strength of the solidified sludge were 451 KPa, 712 KPa, 762 KPa, 838 KPa, and 1064 KPa, respectively. Under 20°C curing conditions, the UCS of the cement-solidified sludge was greater than that of sample F4G6 and F2G8. The 28 days and 90 days strengths of sample F4G6 at 40°C were 1089 KPa and 1169KPa, respectively, which were 1.3 times and 1.1 times that of pure cement curing agent. The 28 days and 90 days strengths of sample F4G6 at 60°C were 1199 KPa and 1282 KPa, respectively, 1.4 times and 1.2 times that of pure cement curing agent. The 28 days and 90 days strengths of F2G8 at 40°C were 1139 KPa and 1194 KPa, respectively, 1.4 times and 1.1 times that of pure cement curing agent. The strengths of sample F2G8 at 14、28 and 90 days curing temperature of 60°C were 802KPa, 1298KPa and 1363KPa, respectively, 1.1 times,1.5 times and 1.3 times that of pure cement curing agent. Due to the rapid hydration reaction rate of cement solidified sludge at room temperature and the slow chemical reaction rate of alkali-activated cementitious materials [28], the strength of samples F4G6 and F2G8 were always less than that of cement solidified sludge at 20°C curing conditions. the hydration reaction and pozzolanic reaction of alkali-activated cementitious materials were accelerated with higher temperature, and a large number of gel products were produced, which played a bonding role between particles. Under the action of alkaline environment, the silicon-aluminum grid structure continued to form and connect with each other to form a denser network structure with the development of curing age, so that the compressive strength of lime-activated industrial waste was continuously enhanced. Therefore, under the high temperature effect, lime-activated fly ash-slag solidified sludge could significantly improve the mid-late strength of the sample.

### Micro-mechanism analysis

#### SEM analysis

**Fig 4:**
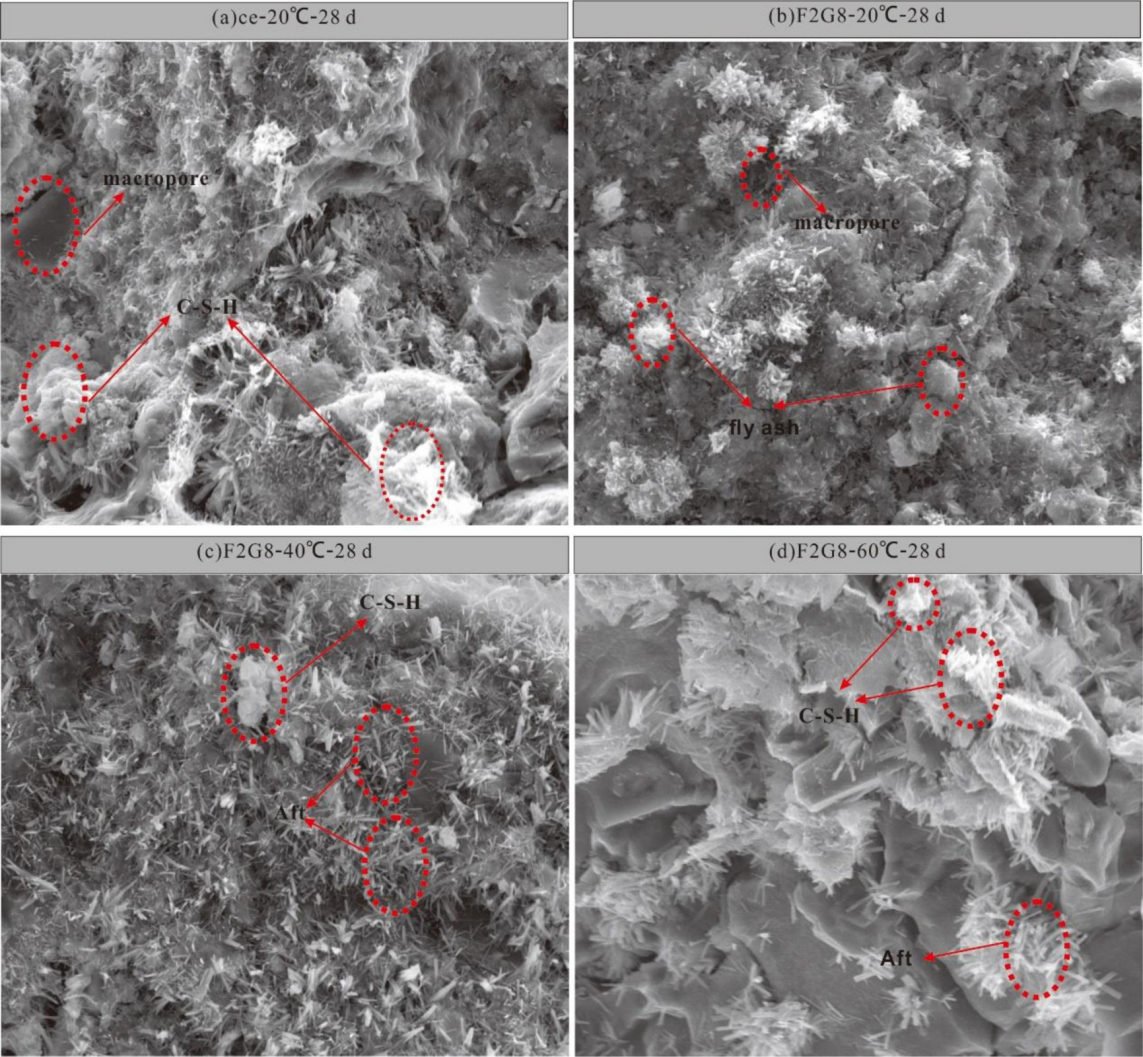
SEM images of cement solidified sludge samples and F2G8 at 28 d of curing under different curing temperature conditions. ce-20°C -28d represents the SEM images of pure cement solidified sludge samples cured to 28 days at 20°C. The meaning of other symbols and so on. Macropore represents the microscopic gap between substances; C-S-H represents calcium silicate hydrate; Aft represents ettringite.

The pore structure and microstructure of lime-activated fly ash-slag solidified sludge were observed and verified by SEM test. Fig 4(A) presents a SEM image of cement solidified sludge after 28 days of curing, which was magnified by 2000 times. Fig 4(B)–4(D) exhibit the SEM images of the representative sample F2G8 enlarged by 2000 times after 28 days of curing. As shown in Fig 4(A), at 20°C, C-S-H and other gel substances were observed in the cement solidified sludge sample, indicating the occurrence of the hydration reaction of cement; the spherical fly ash particles were also found, as shown in Fig 4(B). At this time, the fly ash did not react at room temperature, the gap between the particles was large, and the UCS of the specimen was low. The temperature rised to 40°C, the surface of soil particles showed obvious erosion damage, when, C-S-H and other substances of amorphous gel phase were observed in SEM images, and a large number of needle-shaped rod ettringite were found. These gel substances would be eroded by fly ash, slag and soil particles to form a whole. As the curing temperature rose to 60°C, more amorphous gels (such as C-S-H and Aft) were produced after further activation of slag and fly ash, indicating that the alkaline environment provided by the hydration reaction of lime stimulated the activity of slag and fly ash under the high temperature effect. The generated gel material could better agglomerate the particles and fill the pores between the particles, so that the overall structure of the solidified sludge was more compact, and the strength was higher, consistent with the strength analysis results.

#### XRD analysis

**Fig 5:**
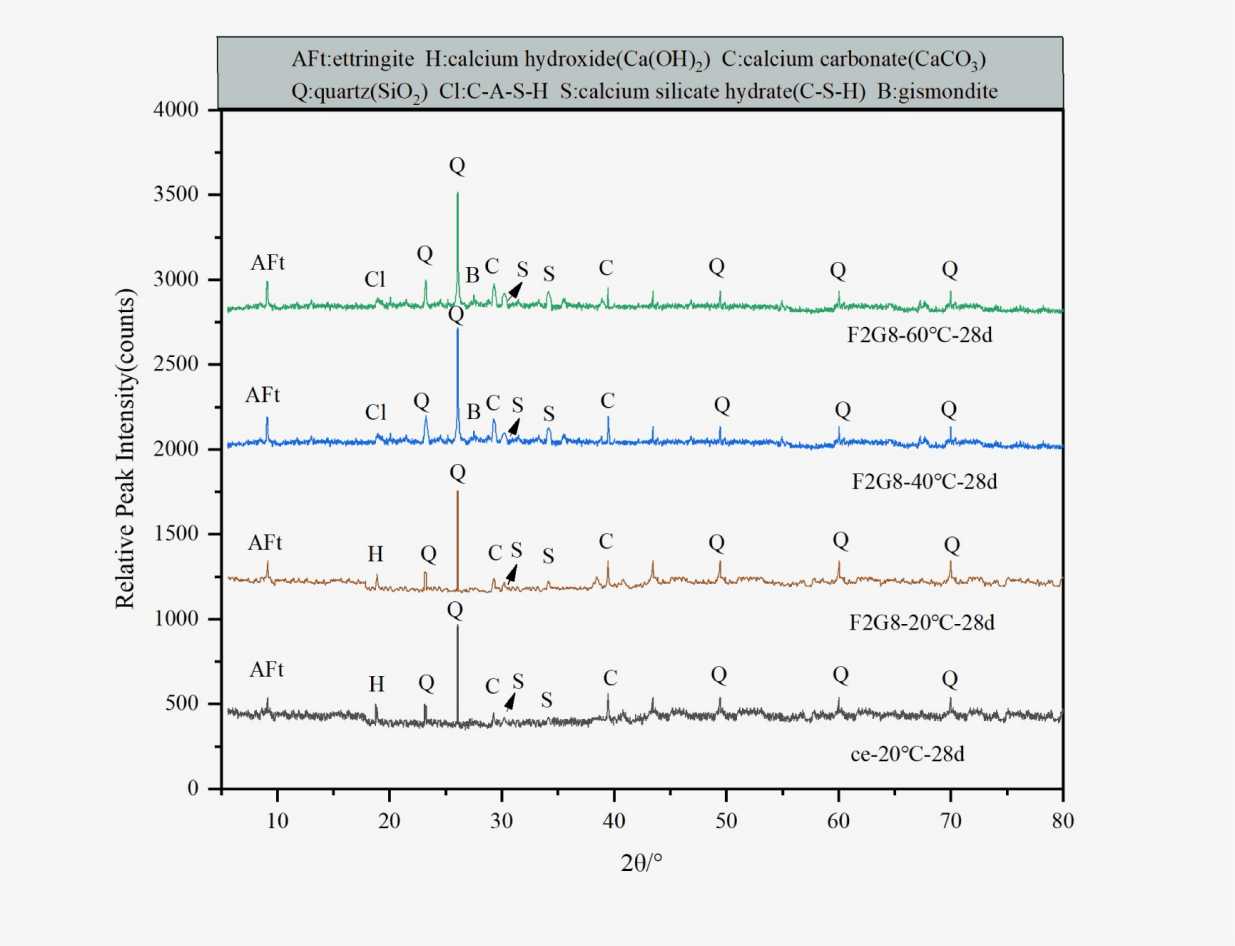
XRD test results of cement solidified sludge samples and F2G8 solidified sludge samples. The mineral composition content of the material was quantitatively analyzed by XRD. (data presented in S4 Table in S1 File).

In order to find out the composition of hydration products of lime-activated fly ash-slag solidified sludge in high temperature environments, XRD test was carried out on the representative sample F2G8 of 28 d curing age. The XRD patterns of cement solidified sludge samples and F2G8 solidified sludge samples are shown in Fig 5. Gel products could be obviously observed in the region of 2*θ* < 45°. With the increase of curing temperature, the diffraction peaks such as calcium hydroxide in the curing agent disappeared, reflecting the chemical reaction between the substances and the formation of new substances. CaO in lime, SiO_2_ and Al_2_O_3_ in fly ash and slag could form ettringite under certain conditions [29], and ettringite could be formed more easily under the condition of high initial water content, filling the pores. The quartz diffraction peak in the 60°C sample was generally lower than that in the 20°C sample, but the diffraction peak of C-S-H in the 60°C sample was slightly higher. This suggested that under high-temperature curing and alkaline conditions, fly ash and slag exhibit increased activity, leading to the hydration of SiO_2_ and Al_2_O_3_, ultimately forming C-S-H gel products. Under the combined influence of high temperature and water medium, lime effectively stimulated the potential active components between fly ash and slag, breaking the Al-O bond and Si-O bond, and repolymerizing to form gel products, which further promoted the improvement of the UCS of sludge under high temperature curing. Furthermore, the diffraction peak of hydrocalumite (CaAl_2_Si_2_O_8_·4H_2_O) was also detected in the solidified sludge, which was derived from the hydration reaction of active SiO_2_ and Al_2_O_3_ with Ca(OH)_2_ [30], and also played a cementing role in the solidified sludge.

#### Summary

Under the influence of high temperature, the influence of new compound curing agent on curing sludge was hereby explored. The following discussions were carried out from the three aspects of curing agent content, curing temperature and curing age: (1) High temperature curing proved to be effective in accelerating the hydration reaction between raw materials. The gel generated by the chemical reaction filled the gap between the particles, thereby improving the strength of the solidified sludge. (2) This paper specifically examined the variation of UCS of samples as curing temperature and curing age increased when adding a 10% curing agent. A parallel comparison test was conducted with cement-solidified sludge samples cured at 20°C. The findings indicated the potential of the new mixed curing agent to partially replace the cement curing agent. Future research should concentrate on exploring alternatives with a higher proportion of curing agents. (3) Under the condition of high temperature curing, more C-S-H and Aft were produced after the further activation of slag and fly ash, which indicated that the alkaline environment provided by the hydration reaction of lime stimulated the activity of slag and fly ash in high temperature environments. The generated gel material could better agglomerate the particles and fill the pores between the particles, so that the overall structure of the solidified sludge was more dense, and the strength was higher. Subsequently, the influence mechanism of blast furnace waste heat on lime-fly ash-slag mixed curing agent should be further studied.

## Conclusions

In this experiment, the UCS and microstructure of sludge solidified by a new composite curing agent were studied, taking into account the effect of curing temperatures on the sludge curing system. The effects of fly ash dosage, slag dosage, age of curing and curing temperature on the development of sludge strength and the microevolution mechanism were systematically investigated by UCS, SEM and XRD experiments. The following are the primary conclusions:

(1) In high-temperature conditions, lime proved effective in stimulating hydration and pozzolanic reactions among fly ash, slag, and sludge. This very stimulation was advantageous for enhancing both the early and long-term strength of the sample. Furthermore, under alkaline conditions, the reaction of hydroxide ions with silica and other substances resulted in the formation of a substantial C-S-H gel. Part of the slag and fly ash were eroded to release free silicon-aluminum substances, and then reacted with Ca^2+^ and SO_4_^2-^ to form needle-like ettringite. The gel generated by the chemical reaction filled the gap between the particles, consequently improving the overall strength of the solidified sludge.

(2) At a curing temperature of 20°C, the strength of pure cement solidified sludge surpassed that of other samples. At 40°C, the 28 days and 90 days strengths of the sludge samples with 20% fly ash doping and 80% slag doping were 1,139 KPa and 1,194 KPa, respectively, which were 1.4 and 1.1 times of the strength of the pure cement-cured sludge; and when the curing temperature was 60°C, the 14, 28 and 90 days strengths of the samples were 802 KPa, 1,298 KPa and 1,363 KPa, respectively, which were 1.1 times, 1.5 times and 1.3 times of the strength of the pure cement-cured sludge. This indicated that lime-fly ash-slag mixed curing agent could substitute cement curing agent partially under high temperature effect.

(3) The improvement of the lime-fly ash-slag solidification sludge was significantly influenced by high temperature curing. In the actual construction process, high temperature climate could be appropriately selected for construction and curing, and the waste heat of blast furnace could be used to enhance the sludge solidification process and lower project costs, so as to achieve the purpose of waste treatment and sustainable development.

## Supporting information

S1 File Supporting data. (XLS)
